# Boosting Electrochemical Performance via Extra‐Role of La‐Doped CeO_2‐δ_ Interlayer for “Oxygen Provider” at High‐Current SOFC Operation

**DOI:** 10.1002/advs.202402348

**Published:** 2024-09-27

**Authors:** Xuan Dong Nguyen, Sang Won Lee, Su Ji Kim, Jungdeok Park, Bonseok Koo, Seok Hee Lee, Shiwoo Lee, Hyung Tae Lim, John T.S. Irvine, Tae Ho Shin

**Affiliations:** ^1^ Korea Institute of Ceramic Engineering and Technology (KICET) Gyongsangnam‐do Jinju‐Si 52851 Republic of Korea; ^2^ Department of Materials Convergence and System Engineering Changwon National University Changwon 51140 Republic of Korea; ^3^ Samsung Electro‐Mechanics Co. Ltd 150, Maeyoung‐ro (Maetan‐dong), Yeongtong‐gu, Suwon‐si Gyeonggi‐do 16674 Republic of Korea; ^4^ School of Chemistry University of St Andrews St Andrews Fife KY16 9ST UK

**Keywords:** lanthanum‐doped ceria, oxygen partial pressure, oxygen storage capacitance, solid oxide fuel cells, starving conditions

## Abstract

Utilizing rare earth doped ceria in solid oxide cells (SOCs) engineering is indeed a strategy aimed at enhancing the electrochemical devices' durability and activity. Particularly, Gd‐doped ceria (GDC) is actively used for barrier layer and catalytic additives in solid oxide fuel cells (SOFCs). In this study, experiments are conducted with La‐doped CeO_2_ (LDC), in which the Ce sites are predominantly occupied by La, to prevent the formation of the Ce‐Zr solid solution. This LDC is comparably used as a functional interlayer between the electrolyte and cathode if sintered at lower temperatures to avoid La_2_Zr_2_O_7_ impurity. In addition, the high substitution of La^3+^ into the ceria lattice improves the oxygen non‐stoichiometry of LDC, leading to accelerated electrochemical high performance by the additional role of LDC for oxygen supplier capacitance at high current operation. Thus, it is confirmed that the improved SOFC high performance is achieved at the maximum power density (MPD) of ≈2.15 W cm^−2^ at 800 °C when the optimized LDC buffer layer is hired at the anode‐supported typed‐Samsung's SOFC by lowering the sintering temperature to prevent LDC's impurity reaction.

## Graphical abstract

The extra role of the La‐doped ceria cathode interlayer as an “oxygen provider” is investigated by measuring the oxygen partial pressure and chemical capacitance at the cathode/electrolyte interface. The high oxygen storage capacitance significantly boosts the electrochemical performance during high current SOFC operation.

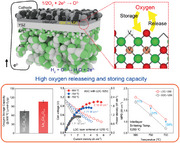

## 1 Introduction

Solid oxide fuel cell (SOFC) is one of the most attractive electrochemical devices owing to its high energy efficiency, ecofriendly power generation with low emission, and fuel flexibility.^[^ ^1^, ^2^ ^]^ However, there are several challenging issues to be solved: cost‐effective manufacturing configuration, lowering the operating temperature, and durability. Developing novel materials or long‐lasting electrode/electrolyte interfaces that permit high activity (oxygen reduction or fuel oxidation) while maintaining long‐term stability is one of the major barriers to the commercialization of the SOFC. Moreover, air utilization efficiency seriously affects the electrochemical performance drops and cell degradation from the viewpoint of the cathode working as the momentary oxygen‐starving atmosphere at a high current range.^[^ ^3^ ^]^ Therefore, developing oxygen electrode materials and interfacial structures with decent reaction activity and stability for oxygen reduction reactions (ORR) is highly desirable. Developing SOFC cathodic layers should be considered in terms of a variety of factors, including i) the decomposition of cathode materials;^[^ ^4^, ^5^, ^6^ ^]^ ii) the chemical incompatibility with electrolytes during manufacturing processes;^[^ ^7^, ^8^, ^9^, ^10^ ^]^ iii) the declining oxygen molecule concentration at the interdiffusion layer of the cathode side. Chemical compatibility between the hierarchical interface components in SOFCs is an emerging critical issue that significantly impacts the durability of SOFCs.^[^ ^11^, ^12^, ^13^, ^14^ ^]^

In SOFC engineering, rare earth doped ceria plays a crucial role in redeeming both durability and performance to overcome chemical compatibility issues. Among these, Gd‐doped ceria (GDC) stands out as a preferred choice due to its dual functionality: it serves as an effective barrier layer to prevent impurity reactions and acts as a catalytic additive, enhancing the efficiency of triple phase boundary (TPB) sites with yttria‐stabilized ZrO_2_ (YSZ) electrolyte.^[^ ^13^, ^15^, ^16^ ^]^ Despite its advantages, the high temperature sintering process of GDC presents unresolved challenges, such as the diffusion of cerium (Ce) atoms into the lattice of ZrO_2_ of YSZ‐based electrolyte, leading to the formation of solid solution. Furthermore, there is a tendency for gadolinium ions (Gd^3+^) to migrate from their initial lattice sites, resulting in the formation of a complex quaternary oxide compound of (Zr, Ce, Gd, Y) O_2_.^[^ ^17^, ^18^, ^19^, ^20^, ^21^ ^]^

In contrast, La‐doped CeO_2−δ_ (LDC) is rarely employed as a buffer layer in YSZ electrolyte systems because it has lower conductivity than GDC; in addition, La content reacts with Zr producing interfacial insulating layers during high‐temperature sintering process, such as La_2_Zr_2_O_7_ impurities, on the YSZ electrolyte. However, LDC presents a promising alternative, contingent upon the absence of impurity reactions. In such scenarios, lanthanum (La) atoms can predominantly occupy Ce sites within the CeO_2−δ_ lattice, thereby enhancing oxygen non‐stoichiometry and significantly increasing oxygen vacancy concentration. Recently, H. Sumi et al. reported that a La_0.1_Ce_0.9_O_2−δ_ (LDC10) interlayer with YSZ electrolyte has lower resistance than the GDC interlayer resulting in slightly improved power density, despite the lower conductivity of LDC10.^[^ ^22^ ^]^ Although LDC has originally lower conductivity than GDC, there might be no serious difference in the conductivity between LDC and GDC when they are inserted as a functional buffer layer because of the Ce‐Zr‐Gd solid solution phase and porosity rather than that of the original GDC layer. It might be evident that LDC offers an extra potential role compared to GDC, particularly in mitigating oxygen starving conditions at high current operation.

Therefore, the significance of selecting a structure or materials that ensure seamless oxygen mobility within the cathode and interfacial layers could not be overstated. When it comes to oxygen‐starving conditions and ORR, high oxygen storage capacitance (OSC) of the material components at the fuel cell cathode might be one of the sensible strategies to improve the stability and performance of the cathode. The efficient oxygen molecules supply seriously affects cell performance and durability from the viewpoint of the ORR/OER (oxygen evolution reaction) working on the electrodes.^[^ ^23^ ^]^ The high OSC interface between electrode and electrolyte might effectively respond to continuously supplying interstitial oxide ions because it can release oxygen from its lattice storage to the surface or bulk when oxygen molecular starving conditions occur in high current operating processes.

In this study, we, herein, highlight a novel aspect of “oxygen provider” and refocus on LDC, emphasizing its capacity as a superior oxygen supplier with greater oxygen storage capability than GDC. This advantage becomes particularly significant when LDC is utilized as a conventional buffer layer for SOCs, a process optimized by lowering the sintering temperature to avoid La_2_Zr_2_O_7_ impurity reaction. This study indicates that the optimized LDC layer, fabricated to avoid impurity reactions, functions as an effective oxygen reservoir. It consistently supplies oxide ions under oxygen‐deficient conditions during operation. This role is attributed to its capacity to enhance the oxide ion supply at the cathode/electrolyte interface, significantly improving superior performance at high current range conditions typical of practical SOFC applications.

## 2 Result and Discussion

**Figure 1:**
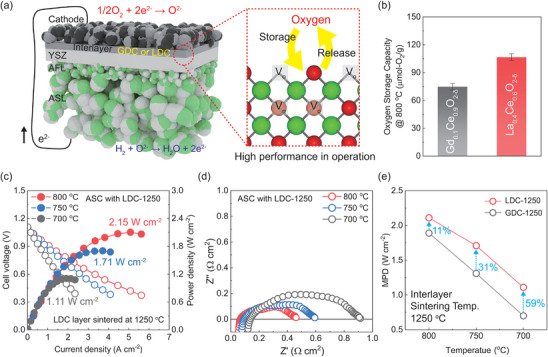
a) Schematic illustration of the single cell and electrochemical behavior of the high OSC layer, enabling high performance in operation. b) The OSC of fluorite structured GDC and LDC at 800 °C. c) *I–V* curves of the optimized anode‐supported cell (ASC) using the LDC layer. d) Electrochemical impedance spectra (EIS) of the optimized ASC with LDC layer. e) Comparison of cell performance with different GDC and LDC layers.

In the SOFC system, where oxygen ion circulation is integral across all components, we hypothesized that selecting materials with high OSC would enhance the overall cell performance as conceptualized in **Figure** **1**a. The LDC layer, exhibiting superior OSC compared to GDC layer (Figure 1b), was optimized by sintering at 1250 °C in an anode supported cell (ASC). Electrochemically, the ASC with the optimized LDC buffer layer achieved remarkable maximum power densities (MPDs) of 2.15, 1.71, and 1.11 W cm^−2^ at 800, 750, and 700 °C, respectively and low total resistances of 0.45, 0.6, and 0.92 Ω cm^2^ as depicted in Figure 1c,d. In comparison to the GDC layer, known for its high conductivity yet lower OSC, the introduction of LDC layers markedly improved performance, showing a 59% increase at the lower temperature of 700 °C, as evidenced in Figure 1e. Consequently, outcomes underscore the pivotal role of OSC in the interlayer for optimizing oxygen ion transport within the cell, thereby significantly boosting operational performance. We will progressively describe the more specific basis, as shown in the following results and discussions.

**Figure 2:**
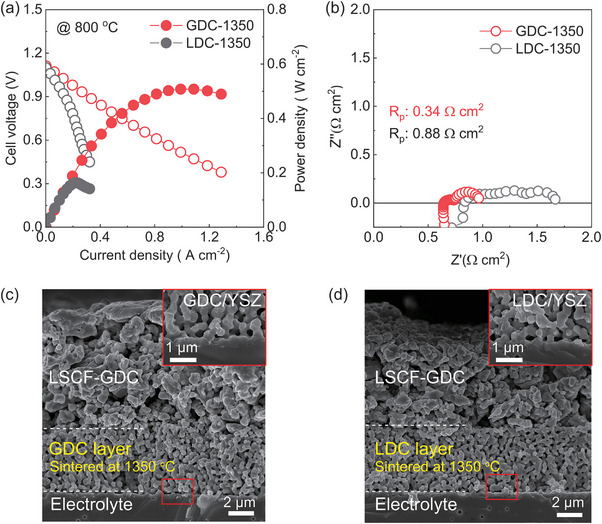
a) *I–V* curves for the electrolyte‐supported cell (ESC) using GDC or LDC layer sintered at 1350 °C. b) EIS results of the cells under OCV conditions at 800 °C. c,d) SEM images of a cell using GDC and LDC layer sintered at 1350 °C.

First, to understand why LDC was rarely used for the barrier layer on the YSZ electrolyte, we compared the performance of LDC and GDC in the conventional sintering process. **Figure** **2**a presents the *I–V* curves of these two electrolyte supported cells (ESC), including the GDC cell sintered at 1350 °C (GDC‐1350), and the LDC cell sintered at 1350 °C (LDC‐1350). The maximum power density (MPD) of the GDC‐1350 was ≈0.45 W cm^−2^ as opposed to the MPD of the LDC‐1350 was only 0.16 W cm^−2^ at 800 °C. The variation in results could be attributed to the formation of La_2_Zr_2_O_7_ of LDC compared to GDC as a result of impurity reactions.^[^ ^22^, ^24^, ^25^ ^]^ Figure 2b shows the EIS results of the LDC‐1350 and GDC‐1350. The ohmic resistance (*R*_s_) is represented as the intercept of the impedance semicircle with the real axis in the high frequency, which approximates the sum of the ohmic resistance of the layers of each component, with contributions mainly from the YSZ electrolyte and interlayer. The cell configurations employ the same YSZ electrolyte and NiO cermet anode, each ≈150 and 20 µm thick, respectively (Figure S1, Supporting Information). Meanwhile, in the low frequency, the intercept of the impedance with the real axis indicates the non‐ohmic resistance, with the difference between the two intercepts representing the polarization resistance (*R*_p_) contributing electrochemical reaction of the electrode. The *R*_s_ as well as *R*_p_ of LDC‐1350 (*R*_p_, 0.88 Ω cm^2^) are more than twice those of GDC‐1350 (*R*_p_, 0.34 Ω cm^2^), which is probably related to the impurities under high‐temperature sintering, 1350 °C. GDC‐1350 achieved a reasonable *R*_p_ value; nevertheless, the interfacial layer formation between the GDC/YSZ bilayer (≥ 1300 °C) has been observed.^[^ ^10^, ^26^, ^27^ ^]^ This observation implies that the formation of an interdiffusion layer between the rare earth doped ceria interlayer and the YSZ electrolyte during high‐temperature processing (above 1300 °C) may restrict the electrochemical performance of the system.^[^ ^21^, ^27^, ^28^, ^29^ ^]^ LDC has rarely been chosen as a less favored choice among these options because of lower performance and the severe impurities reaction during high temperature processing.

**Figure 3:**
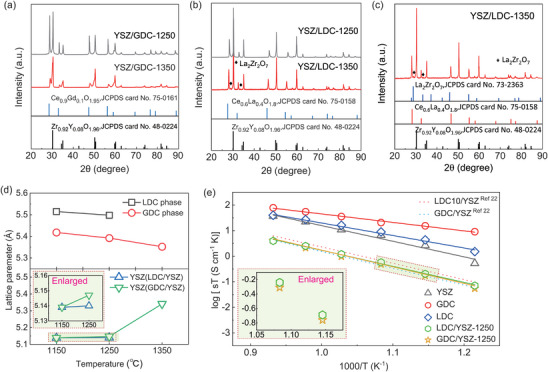
a,b) XRD patterns of GDC/YSZ and LDC/YSZ composite sintered at 1250 and 1350 °C. c) Enlarged XRD pattern of the LDC/YSZ composite sintered at 1350 °C. d) Lattice parameter from Rietveld refinement of LDC and GDC phase in the composite. e) Comparison of the conductivity of the GDC, LDC, YSZ, GDC/YSZ, and LDC/YSZ composite.

To comprehend what happened to the interdiffusion layer and chemical compatibility between LDC and GDC with YSZ electrolyte, we conducted a series of X‐Ray diffraction (XRD) analyses on mixtures of LDC and GDC with YSZ powder, sintered at different temperatures. These analyses were visually represented in **Figure** **3**. Specifically, we examined XRD patterns for LDC/YSZ and GDC/YSZ composites sintered at 1250 and 1350 °C, labeled as LDC/YSZ‐1250, LDC/YSZ‐1350, GDC/YSZ‐1250, and GDC/YSZ‐1350, for clarity. The XRD patterns for the GDC/YSZ composites were identified as fluorite structures, showing no significant secondary peaks (as shown in Figure 3a). However, an anomaly was observed in the broadening of peaks for GDC/YSZ‐1350 compared to GDC/YSZ‐1250, indicating the presence of a secondary phase. This observation is further supported by the lattice parameter increase of YSZ mixed with GDC after sintering at 1350 °C, as depicted in Figure 3d (bottom). This suggests that a CeO_2_‐ZrO_2_ solid solution began to form at ≈1350 °C, negatively impacting the potential for achieving optimal SOFC performance due to the detrimental effects of this solid solution formation on the system.^[^ ^13^, ^33^ ^]^ Whilst the XRD analyses of LDC/YSZ‐1250 composite mixture show that no serious secondary reaction occurred below 1250 °C, as shown in Figure 3b. However, for the LDC/YSZ‐1350 composite, the impurity diffraction peaks were observed (Figure 3c), likely indicative of the formation of the pyrochlore‐type La_2_Zr_2_O_7_.^[^ ^34^, ^35^ ^]^ This observation aligns well with the electrical resistance (*R*_s_) and polarization resistance (*R*_p_) measurements for LDC‐1350, as illustrated in Figure 2b.

The lattice parameters of LDC and GDC in the YSZ composite mixtures decrease as depicted in Figure 3d, Figure S2, and Table S1 (Supporting Information). This reduction in lattice parameters might be attributed to the smaller Y^3+^ and Zr^4+^ ions from YSZ occupying Ce sites during heat treatment.^[^ ^29^, ^30^ ^]^ Conversely, the lattice parameter of YSZ expands with higher sintering temperatures in GDC/YSZ‐1350, which might reflect the diffusion of larger Gd^3+^, and Ce^4+^ ions. As the sintering temperature increases, the change in lattice parameters for the GDC/YSZ composite is slightly more pronounced than in the LDC/YSZ composite. This variation may suggest potentially better chemical compatibility between LDC and YSZ under lower temperature (< 1250 °C) sintering conditions.

Figure 3e showcases a comparative analysis of the electrical conductivity among LDC/YSZ‐1250, GDC/YSZ‐1250, GDC/YSZ‐ref, and LDC10/YSZ‐ref samples.^[^ ^22^ ^]^ As shown in Figure 3e, the conductivity of the LDC/YSZ‐1250 sample is very similar to that of the GDC/YSZ‐1250 sample, even though the fully sintered GDC bulk sample exhibits higher conductivity than that of LDC. This could be attributed to the effect of the porous structure, which might diminish the advantage of higher conductivity in GDC. Moreover, below the 750 °C temperature range, the conductivity of the LDC/YSZ‐1250 sample is negligibly yet marginally higher than that of the GDC mixture, as illustrated in the magnified section of the conductivity graph in the lower left corner of Figure 3e. The lower conductivity of the GDC mixture (GDC/YSZ‐1250) than expected might be explained by the formation of an inter‐diffusion layer between GDC and YSZ, facilitated by a Ce and Zr solid solution. In the case of GDC and YSZ, Gd^3+^ (1.01 Å) and Ce are more likely to diffuse into Zr sites. Evidently, the lattice volume and parameter of GDC show an increase as a function of the sintering temperature with the YSZ mixture. In contrast, those of LDC exhibit negligible change up to 1250 °C due to the avoidance of La_2_Zr_2_O_7_ impurity formation below 1250 °C processing. Particularly, the change in the lattice parameter of YSZ (GDC/YSZ) is marginally higher than that of YSZ (LDC/YSZ) with increasing temperature, as shown in a zoom‐in section (the lower left corner of Figure 3d). This suggests that in solid oxide fuel cells (SOFCs), where the functional buffer layer consists of both a porous part and an interdiffusion layer affecting ohmic and polarization resistance, the conductivity of the bulk material might not be as influential as the properties of the interdiffusion impurity layer. Thus, LDC on YSZ could be hired as a conventional buffer layer for SOFCs, provided the process is optimized by reducing the sintering temperature to avoid the La_2_Zr_2_O_7_ impurity reaction.

**Figure 4:**
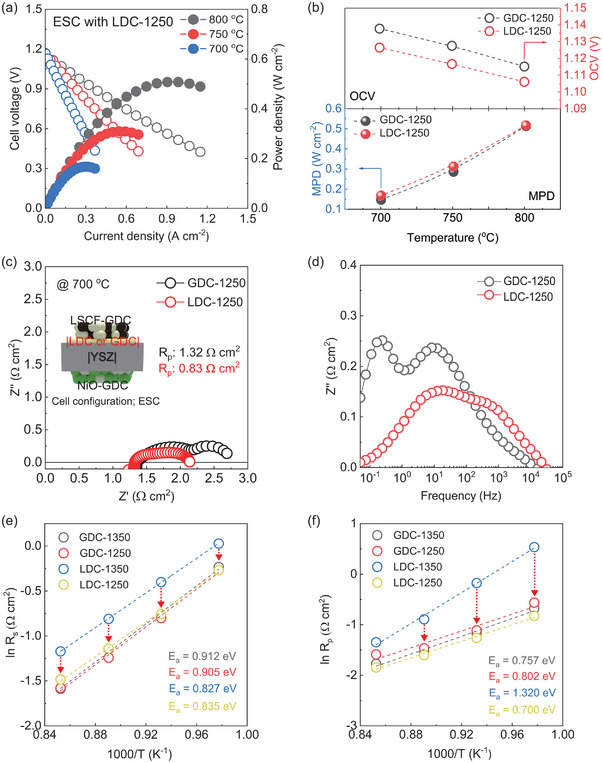
a) *I–V* curves for the ESC cell using LDC layer sintered at 1250 °C. b) OCV and MPD for the cell using GDC and LDC layer fabricated at 1250 °C as a function of temperature. c) EIS results of the cells under OCV at 700 °C. d) Bode plots of the cells at 700 °C. e,f) Arrhenius plots of the *R*_s_ and *R*_p_.

To gain deeper insights into the electrochemical behavior within the GDC and LDC interlayers, we utilized a simple electrolyte‐supported‐button cell (ESC) setup. In **Figure** **4**a,b and Figures S3 and S4 (Supporting Information), the ESC cells incorporating the LDC and GDC sintered at 1250 °C (denoted as LDC‐1250 and GDC‐1250, respectively) were tested. The results showed that at 800 °C, the LDC‐1250 indicates a power density of ≈ 0.51 W cm^−2^, slightly higher than that of the GDC‐1250 with a value of ≈ 0.50 W cm^−2^. Furthermore, at lower temperatures of 750 and 700 °C, the performance gap widened, with the LDC‐1250 (0.31 and 0.18 W cm^−2^) overcome the values of the GDC‐1250 (≈0.28 and ≈0.14 W cm^−2^), as detailed in Table S2 (Supporting Information). Consequently, the electrochemical performance of the LDC‐1250 cell was higher than that of GDC‐1250 cell (Figure 4b).

As shown in Figure 4c, these *R*_s_ values of the two cells are almost the same, which is a good agreement with the conductivity of the YSZ/LDC‐1250 and YSZ/GDC‐1250 interdiffusion layer, as mentioned in Figure 3e. The *R*_p_ of the GDC‐1250 cell is almost 1.5 times larger than that of the LDC‐1250 cell, which is 1.32 and 0.83 Ω cm^2^, respectively. It might be indicated that the more improved performance of the LDC cell outperformed GDC, primarily attributed to reactions involving *R*_p_ impedance which includes the electrochemical capacitance related to oxygen diffusion mobility in the hierarchical interface of SOFCs. Furthermore, the difference from these *R*_p_ mainly occurred in low‐frequency and medium‐frequency characteristic processes, as depicted in Figure 4d, which can be attributed to the electrochemical capacitance, oxygen surface exchange, and oxygen diffusion in the cathode.^[^ ^31^, ^32^ ^]^ Therefore, the distinct *R*_p_ values between the GDC‐1250 and LDC‐1250 cells might hypothesize that this variation is due to an oxygen ion storage capacitance layer at the cathodic interface as an oxygen provider role, which influences the electrochemical reactions and oxygen transport dynamics.

**Figure 5:**
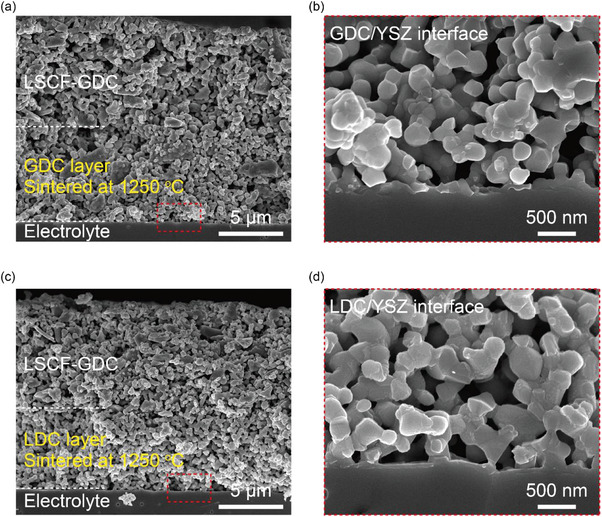
SEM images for the cross–section of the cell containing a,b) GDC and c,d) LDC interlayer sintered at 1250 °C.

The Arrhenius plots in Figure 4e,f shows the activation energy of *R*_s_ and *R*_p_ of GDC‐1350, LDC‐1350, GDC‐1250, and LDC‐1250. The *R*_s_ and *R*_p_ activation energy of these cells fabricated at 1350 °C are higher than those of the other, and this might have occurred because of the existence of the CeO_2_‐ZrO_2_ solid solution or La_2_Zr_2_O_7_. Reducing the sintering temperature of the LDC layer to 1250 °C leads to a marked reduction in both *R*_s_ and *R*_p_. Additionally, the LDC‐1250 shows the lowest activation energy for *R*_p_, recorded at 0.70 eV. The microstructural analysis confirmed that there were no significant differences in pore size or layer thickness between the GDC‐1250 and LDC‐1250 cells as shown in **Figure** **5**. This suggests that the observed variations in resistance *R*_p_ are likely attributable to the intrinsic material properties.

The mid‐term stability of the LDC‐1250 cell was assessed and detailed in Figure S5 (Supporting Information). Tested under 400 mA cm^−2^ current density in H_2_ and air at 750 °C, the cell exhibited a voltage degradation rate of 0.75% over 100 h. This rate of degradation is on par with that observed in cells using a conventional GDC cathode interlayer, as reported in the literature.^[^ ^33^, ^36^, ^37^ ^]^ This comparison underscores the viability of LDC‐1250 as an effective alternative to GDC, maintaining comparable durability under operational conditions.

**Figure 6:**
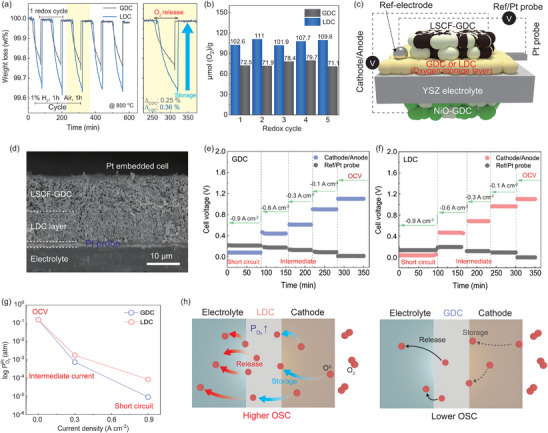
a) Thermal gravity analysis (TGA) of during redox cycle GDC and LDC exposed to air and 1% H_2_ repeatedly for 1 h. b) OSC value of GDC and LDC under redox 5 cycles. c) A schematic of an ESC with a Pt probe in the buffer layer on YSZ electrolyte. d) SEM image of the Pt‐embedded cell. e,f) Electric potential change of GDC and LDC cell during the applied current. g) The estimated PO_2_ in the interface of GDC/YSZ and LDC/YSZ under open circuit, intermediate load, and short circuit conditions. h) A schematic describes the OSC of these interlayers of LDC and GDC layers.

To further demonstrate that the oxide ion‐releasing and storing capacity electrochemically contributes to the enhancement of the SOFC performance, a thermal gravity analysis (TGA) of the LDC and GDC was conducted. The oxygen storage capacitance (OSC) of GDC and LDC was evaluated through the measurement of weight loss (%) during redox cycles, as illustrated in **Figure** **6**a,b. The OSC of the LDC was found to be greater than that of GDC by ≈1.5 times, which is ≈111 and 72.5 µmol (O_2_/g), respectively. This is a good agreement of the higher performance of the cell incorporating an LDC buffer layer, which was fabricated at 1250 °C sintering without La_2_Zr_2_O_7_ impurity reaction, than that of GDC at the high current operation as shown in Figure 4a. This emphasizes that the electrochemical performance of SOFCs depends not just on electrical conductivity but also crucially on oxygen storage capacitance (OSC). It highlights OSC's vital contribution to improving the overall efficiency and effectiveness of SOFC high‐current operations. It is well known that La^3+^ (1.13 Å), with a relatively large effective ionic size compared to other rare‐earth elements, creates more oxygen vacancies than Gd^3+^ (1.01 Å) and Sm^3+^ (0.99 Å), suggesting that the oxygen storage‐releasing capacitance of doped ceria is strongly related to the ionic radius.^[^ ^38^ ^]^

To assess how the oxygen storage and supply capabilities of the interlayer affect the stability of oxygen partial pressure and potential at the YSZ electrolyte interface when GDC and LDC buffer layers are employed, relatively. Figure 6c provides a schematic illustration of the Pt‐embedded cell.^[^ ^39^, ^40^, ^41^ ^]^ A Pt probe was embedded between the YSZ electrolyte and the LDC (GDC) layer for counting electric potential at the Pt probe electrode.

Figure 6d and Figure S6 (Supporting Information) present the cross–sectional images of the Pt‐embedded cell after the test. A thin Pt probe was observed between the dense electrolyte and the porous interlayer. The electric potential changes at the YSZ/LDC‐1250 and YSZ/GDC‐1250 interfaces as a function of current density, as shown in Figure 6e,f, respectively. The overpotential of the LDC cell is less than that of GDC, during applied current density, while the different potential between the reference electrode and LDC/YSZ interface is greater than the value of the GDC. Based on the electric potential measurement results, the oxygen partial pressure at the interface of Insert layer/YSZ (oxygen pressure at the Pt probe, $P_{O_{2}}^{Pt}$), can be estimated using Nernst equation:

(1) $$\varphi^{Ref}-\varphi^{Pt}=\frac{RT}{nF}\ln\left(\frac{P_{O_{2}}^{Ref}}{P_{O_{2}}^{Pt}}\right)$$

where *R* is the ideal gas law constant, *T* is absolute temperature, *F* is Faraday's constant, φ^*Pt*^ is the electric potential at the Pt probe, and φ^*Ref*^ is the electric potential at the reference electrode. It was assumed that the oxygen partial pressure of the reference electrode ($P_{O_{2}}^{Ref}$) is constant $P_{O_{2}}^{Ref}$ ≈0.21 atm under any circumstances. Figure 6g shows the current density dependence of oxygen partial pressure at varying conditions. The $P_{O_{2}}^{Pt}$ value has been shown rapidly decrease tendency with increasing current flux, which can be named as the starving condition. At OCV, the $P_{O_{2}}^{Pt}$ is estimated as ≈0.16 atm in both LDC and GDC interlayers. The $P_{O_{2}}^{Pt}$ across the buffer layer/electrolyte interface was slightly reduced compared to the air atmosphere because of the porous interlayer. Compared to a cell made with a fully dense GDC electrolyte in the literature, the estimated $P_{O_{2}}^{Pt}$ was ≈1.23 × 10^−3^ atm.^[^ ^39^, ^40^ ^]^ The $P_{O_{2}}^{Pt}$ associated intermediate current, and short circuit conditions were all determined in the same way. For the intermediate current density (300 mA cm^−2^), the $P_{O_{2}}^{Pt}$ of the GDC and LDC cell were $P_{O_{2}}^{Pt}$ (GDC) ≈7.5 × 10^−4^ atm and $P_{O_{2}}^{Pt}$ (LDC) ≈1.8 × 10^−3^ atm, respectively. It shows $P_{O_{2}}^{Pt}$ (GDC) ≈9.2 × 10^−6^ atm and $P_{O_{2}}^{Pt}$ (LDC) ≈8.4 × 10^−5^ atm at short circuit conditions (900 mA cm^−2^). Generally, these results show the $P_{O_{2}}^{Pt}$ are decreasing from OCV to short circuit conditions. The $P_{O_{2}}^{Pt}$ of LDC and YSZ was greater than that at the interface of GDC and YSZ, which can be attributed to the effect of the high oxygen storage capacity provided by LDC.^[^ ^38^ ^]^ As a result, during the oxygen reduction reaction over the cathode, oxygen ions are stored in the lattice of the LDC buffer layer. The stored oxygen ions can be continuously supplied to the bulk electrolyte, thereby enhancing cell performance. (Figure 6h)

**Figure 7:**
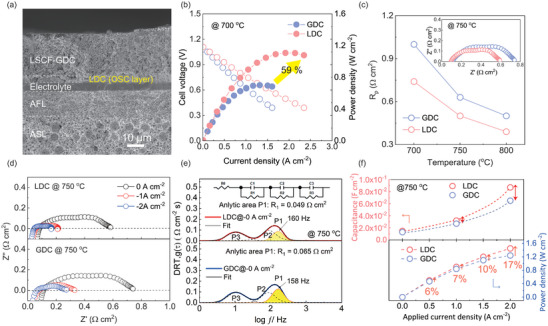
a) Cross–section SEM image of the optimized ASC with LDC‐1250 layer. b) *I–V* curves for the ASC cells with LDC and GDC layer at 700 °C. c) EIS results for the ASC cells with different interlayers. d) EIS dates and e) distribution of relaxation time (DRT) plots of ASC cells under applied current density. f) The dependence of capacitance at the cathode/electrolyte interface and power density on current flux.

In this study, we conclusively verify that the optimized LDC buffer layer maintains higher electrochemical performance as a sufficient oxygen provider during high‐current range operation and achieves superior performance, particularly in anode‐supported cells (ASC). The SEM images of the ASC are shown in **Figures** **7**a and S7 (Supporting Information). A thin and dense YSZ electrolyte layer is sandwiched between the porous oxygen electrode and the fuel electrode. Figure 7b and Figure S8 (Supporting Information) present the electrochemical performance of the ASCs, with the oxygen storage interlayer of LDC and GDC layer at 700 °C. The cell with LDC achieved a high performance of 1.11 W cm^−2^, outperforming that of the GDC cell (0.70 W cm^−2^), caused by the high OSC behavior. Figure 7c and Table S3 (Supporting Information) indicated that *R*_p_ of the LDC over GDC aligns with our prior discussion in ESC results (Figure 4). Moreover, the ohmic resistance (*R*_s_) of the LDC buffer layer, sintered at temperatures below 1250 °C, is marginally lower than that of GDC despite the higher conductivity of GDC compared to LDC. This phenomenon could be attributed to the conductivity of the inter‐diffusion layer between the LDC buffer layer and the YSZ electrolyte, consistent with the results for the mixture conductivity depicted in the magnified graph in Figure 3e. Here, the lower *R*_s_ of the LDC buffer layer further confirms the previously mentioned findings, suggesting that the inter‐diffusion layer between the LDC and the YSZ electrolyte, when sintered at 1250 °C, might exhibit a conductivity that is marginally higher than that of the GDC.

Analyzing the connection between overpotential and *R*_p_ is crucial for understanding the relationship between OSC and SOFC performance. It is also essential for exploring and understanding the supply and flow of oxygen in real time, especially within the range of high currents. Therefore, an electrochemical analysis of the impedance spectra data of ASC cells under current flux was carried out. As shown in Figure 7d, the *R*_p_ for both samples is reduced significantly in current flux conditions, boosting the electrochemical reaction process associated with the electrode's activation.^[^ ^42^, ^43^ ^]^

Moreover, upon the increase of the current density, the LDC (0.15 Ω cm^2^) verifies lower polarization as opposed to the GDC (0.24 Ω cm^2^). This finding highlights the advanced OSC of the LDC, signifying its notable improvement in the comprehensive electrochemical reaction throughout practical cell operation. DRT analysis was collected to investigate the oxygen chemical capacitance of these interlayers under various current conditions, as shown in Figure 7e and Figure S9 (Supporting Information), which revealed major processes in the electrodes. In DRT plots of ASC cells, the P1 and P2 are associated with oxygen surface exchange and oxygen conduction. P3 is linked to the gas conversion reaction, aligning with the literature findings.^[^ ^42^, ^43^ ^]^ However, to clarify the oxygen release from the cathode to the electrolyte, a focused analysis of the P1 characteristics, representing the storage and release of oxygen ions in the interlayer, is essential. It is well known that DRT incorporates R‐CPE parallel circuits (RQ elements) to specify physical‐electrochemical processes during cell testing and accordingly, estimated capacitance (*C_ct_*) regarding the charge transfer of oxygen ions in the electrode/electrolyte interface was derived by P1. The calculation formula of *C_ct_* is expressed as following equation:^[^ ^46^, ^47^ ^]^

(2) $$C_{\mathrm{ct}}=\frac{1}{2\pi f_{\max}R_{\mathrm{ct}}}$$

where *f_max_* is the frequency of the maximum point of the P1. The *R_ct_* is the charge transfer resistance of oxygen ions (P1). The dependence of the chemical capacitance at the interface of cathode/electrolyte on the current density flux is shown in Figure 7f (upper). At the OCV, the LDC cell has a higher *C_ct_* of oxygen ion than that of the GDC. As the applied current increases, the difference in *C_ct_* values between LDC and GDC progressively widen. Moreover, a more pronounced tendency is observed, especially, when the current density is over 2 A cm^−2^. Figure 7f (bottom) shows how power density in SOFCs increases with current density. Particularly, LDC's performance growth surpasses GDC's as current density heightens, a difference that intensifies at higher currents. This key observation highlights the superior rate at which LDC performs under increased current loads compared to GDC. It suggested that we could get high performance of SOFC with LDC because of the high oxygen capacitance at the high current density range. A higher oxygen ions capacitance allows the release of more charge at the cathode/electrolyte interface, thereby enhancing and protecting the overall performance of the SOFC during the practical oxygen‐starvation condition. The comparison of MPD to a similar SOFCs configuration with a ceria‐based buffer layer was previously reported, as summarized in **Table** **1**.^[^ ^48^, ^49^, ^50^, ^51^, ^52^, ^53^, ^54^ ^]^

**Table 1:** Summary of the electrochemical performance of this work and reported in the literature.

Materials				MPD [W cm^−2^]			Reference
Anode	Electrolyte	Interlayer	Cathode	800^o^C	750^o^C	700^o^C	
Ni‐YSZ (tape‐casting)	YSZ (5 µm, tape‐casting)	LDC ≈5 µ*m*, screen printing)	LSCF‐GDC	2.15	1.71	1.11	this work
Ni‐YSZ (tape‐casting)	YSZ (10 µ*m*, tape‐casting)	SDC (≈2 µ*m*, wet spraying)	BSCF‐GDC	1.1	‐	‐	[48]
Ni‐YSZ (tape‐casting)	YSZ (10 µ*m*, tape‐casting)	GDC (≈2 µ*m*, aerosol coating)	LSCF‐GDC	‐	0.54	‐	[49]
Ni‐YSZ (tape‐casting)	YSZ (14 µ*m*, slurry casting)	GDC (7 µ*m*, screen printing)	LSCF ‐GDC	‐	0.696	‐	[50]
Ni‐YSZ (tape‐casting)	YSZ (14 µ*m*, slurry casting)	GDC (7 µ*m*, screen printing)	LSCF‐GDC	‐	1.18	‐	[50]
Ni‐YSZ (tape‐casting)	YSZ (5 µ*m*, tape‐casting)	GDC (∼5 µ*m*, screen printing)	LSCF‐GDC	1.89	1.31	0.7	This work
Ni‐YSZ (Pelletizing)	YSZ (3 µ*m*, spray‐coating)	GDC (7 µ*m*, spray‐coating)	LSCF‐GDC	0.68	0.57	0.41	[51]
Ni‐YSZ (pelletizing)	YSZ (4 µ*m*, sputtering)	GDC (3 µ*m*, sputtering)	LSC‐GDC	1.32	1.04	0.79	[52]
Ni‐YSZ (pelletizing)	YSZ ( spin coating)	GDC ( spin coating)	LSF‐GDC	‐	1	0.8	[53]
Ni‐YSZ (tape‐casting)	YSZ (tape‐casting)	GDC ( screen printing)	LSCF‐GDC/LSCF	‐	0.45	‐	[54]

## 3 Conclusion

To conclude, this study has demonstrated the significant advantage of employing an optimized LDC buffer layer when fabricated by lowering the sintering process (< 1250 °C) to avoid La_2_Zr_2_O_7_ impurity reaction in SOFCs, emphasizing its capacity as a superior oxygen supplier with greater oxygen storage capability than GDC. A novel aspect of “oxygen provider”, supported by TGA, demonstrates that LDC possesses a higher OSC than GDC, significantly boosting the cathode's electrochemical performance in SOFCs. Additionally, the oxygen partial pressure at the LDC‐YSZ interface, as measured by embedding a Pt probe, was ≈2.5 times higher than that at the GDC interface under a current density of 300 mA cm^−2^. This indicates that the oxygen‐releasing and storage capacity of the LDC layer improves performance and ensures uninterrupted oxygen ion supply under oxygen‐starved conditions. This advantage becomes particularly significant when LDC is utilized as a conventional buffer layer for high‐performed ASCs. Consequently, ASC with the optimized LDC buffer layer achieved remarkable MPDs of 2.15, 1.71, and 1.11 W cm^−2^ at 800, 750, and 700 °C, respectively. LDC layers as an extra‐role with “oxygen provider” markedly improved performance at high‐current range operation, showing a 59% increase at the lower temperature of 700 °C compared to GDC. As a promising buffer layer material, LDC stands out as an exceptional oxygen supplier, offering enhanced oxygen storage capabilities compared to GDC, thus significantly enhancing SOFC performance.

## 4 Experimental Section

### Preparation of Powder Materials

Powders of YSZ (Tosoh, Japan), La_0.4_Ce_0.6_O_2−δ_ (LDC) (Kceracell, Korea), Gd_0.1_Ce_0.9_O_2−δ_ (GDC), NiO, and La_0.4_Sr_0.6_Co_0.2_Fe_0.8_O_3‐δ_ (Fuel cell materials, USA) were used in this study. LDC or GDC was used for the buffer layers, YSZ for the electrolyte, Ni‐GDC for the anode, and LSCF‐GDC for the cathode. Powders of 50% YSZ were mixed with 50% GDC or 50% LDC using mortars and pestles, resulting in the formation of composites notated as YSZ/GDC and YSZ/LDC. The mixed powders were then heat‐treated in air at 1250 and 1350 °C for 5 h. Similarly, these powders were compressed and sintered at 1250 °C for 3 h in the air for the purpose of the conductivity test.

### Fabrication of Full Cell and Pt Embedded Cell

Two types of full cells were constructed in the study, i) electrolyte‐supported cells (ESCs) and ii) anode electrode‐supported cells (ASCs). For the ESCs with identical anode interlayer GDC, the cathode interlayer LDC (or GDC) was coated by screen‐printing which is the same method for the symmetric cells. After drying, the 7 mm diameter of NiO‐GDC anode was printed on the GDC anode interlayer, and then these samples were co‐fired for 2 h at 1350 and 1250 °C. The oxygen electrodes (active area 0.5 cm^−2^) were screen‐printed onto the LDC (GDC) interlayer and fired at 1050 °C in the air for 1 h. After firing, the interlayers (LDC or GDC) were found to be ≈5 µm thick and the cathode layers were ≈15 µm thick. The resulting cell configuration was Ni‐GDC|GDC|YSZ| (LDC or GDC) |LSCF‐GDC.

For the ASCs, two anode‐supported cells (ASCs) (20 mm diameter) (Samsung Electro‐Mechanics, Korea) were made to confirm the properties of the LDC and GDC, and the configuration was Ni‐YSZ|YSZ| (LDC or GDC) |LSCF‐GDC. The GDC and LDC buffer layer were sintered at 1250 °C for 2 h.

The 150 µm YSZ electrolyte was coated with a thin and narrow strip of platinum paste (Pt probe). The sample was then heated to remove the binder from the platinum paste. Buffer layers were screen‐printed to cover most of the surface, leaving only a small portion of the platinum strip exposed. The cell was sintered at 1250 °C for 2 h, resulting in a ≈10 µm thick interlayer. Following sintering, the anode (NiO‐GDC) and cathode (LSCF‐GDC) were applied and fired at 1050 °C for 1 h. A platinum wire was attached to the exposed platinum strip and sealed with glass sealant. Additionally, a reference electrode was coated on the LDC (GDC) surface near the edge. The electric potential changes at the YSZ/LDC (GDC) interface were measured as a function of current density relative to the reference electrode.

### Characterization of Interlayers and their Electrochemical Properties

X‐ray diffraction (XRD) measurements were conducted on both the powder and composite pellets using a Bragg‐Brentano diffractometer (RIGAKU, RU300, Japan) with Cu‐K ***α*** wavelength (*λ* = 1.541 Å). The crystal structures were analyzed using the Rietveld refinement program, GSAS II. Microstructural and compositional analyses were carried out on the fabricated electrolyte‐supported cell using a field‐emission scanning electron microscope (FE‐SEM). Thermogravimetric analysis (TGA, NETZSCH GERAETEBAU GmbH, Germany) was performed to investigate the redox property during redox cycling under air and 1% H_2_ conditions. Conductivity measurements were conducted on the sintered pellets using standard four‐terminal DC techniques in the ambient air. The performance of the button cell was assessed under humidified hydrogen and air conditions, with a flow rate of 100 ml min^−1^. Four Pt metal lead lines and two Pt mesh were utilized as the commercial current collectors. Current‐voltage characteristics (*I–V* curves) and impedance spectra were measured within the temperature range of 700–800 °C, using a Potentiostat (VMP‐300, Bio‐logic, France). Resistance measurements were conducted under open‐circuit conditions, spanning a frequency range of ≈500k–0.1 Hz, with a signal amplitude of 10 mA. The same measurement conditions were applied to the Pt‐embedded cells as well. In conducting the DRT analysis, it was validated that the actual (*Z*’(ω)) and imaginary (*Z*’’(ω)) impedances adhere to the Kramers‐Kronig relation.

## Conflict of Interest

The authors declare that they have no conflict of interest.

## Supporting information

Supporting Information

## Data Availability

The data that support the findings of this study are available from the corresponding author upon reasonable request.
