# Supplementary material for: Boosting Electrochemical Performance via Extra‐Role of La‐Doped CeO2‐δ Interlayer for “Oxygen Provider” at High‐Current SOFC Operation
Source: Adv Sci (Weinh). 2024 Sep 27;11(46):2402348. doi: 10.1002/advs.202402348 (PMC11633512; doi:10.1002/advs.202402348)
Supplement: Supplementary file 1 — Supporting Information [file ADVS-11-2402348-s001.docx]

Supporting Information

Boosting Electrochemical Performance via Extra-Role of La-Doped CeO_2-δ_ Interlayer for “Oxygen Provider” at High-Current SOFC Operation

Xuan Dong Nguyen^†^, Sang Won Lee^†^, Su Ji Kim, Jungdeok Park, Bonseok Koo, Seok Hee Lee, Shiwoo Lee, Hyung Tae Lim*, John T.S. Irvine, and Tae Ho Shin*


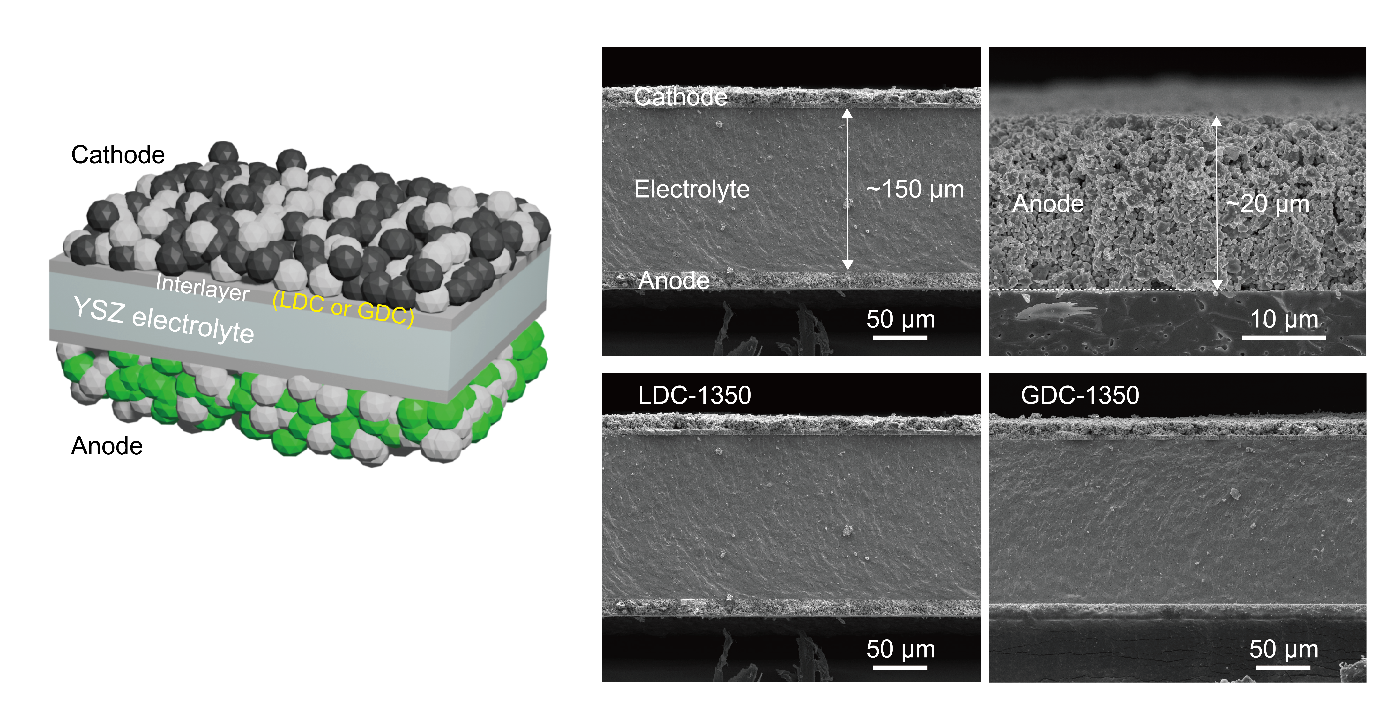


Figure S1. Schematic of the cell configuration of electrolyte-supported cell type and the SEM microstructures of LDC-1350 and GDC-1350


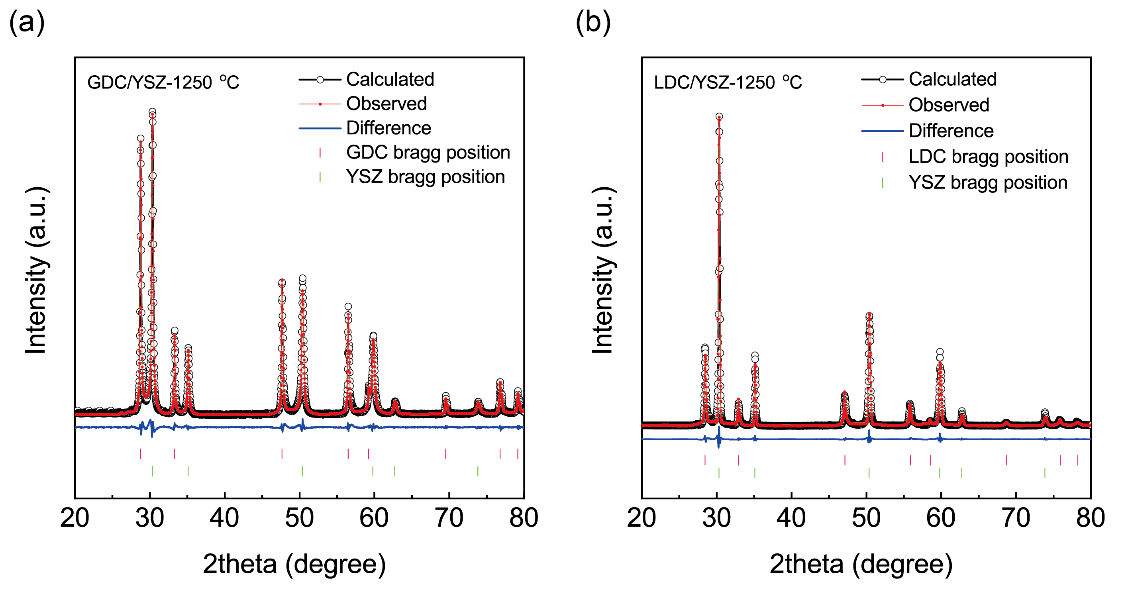


**Figure S2.** The Rietveld refinement profiles from XRD data of (a) GDC/YSZ and (b) LDC/YSZ mixture sintered at 1250 ^o^C.

**Table S1.** Summary of Rietveld Refinement Results for the YSZ, LDC, and GDC powders as a control group and LDC, YSZ in LDC/YSZ composite sintered at 1250 ^o^C, the GDC, YSZ in GDC/YSZ composite sintered at 1250 ^o^C, and the GDC, YSZ in LDC/YSZ composite sintered at 1350 ^o^C.

| Powder |  | YSZ/LDC-1250 | | YSZ/GDC-1250 | | YSZ/GDC-1350 | |
| --- | --- | --- | --- | --- | --- | --- | --- |
| YSZ |  | YSZ phase |  | YSZ phase |  | YSZ phase |  |
| a=b=c (Ȧ) | 5.139 | a=b=c (Ȧ) | 5.142 | a=b=c (Ȧ) | 5.148 | a=b=c (Ȧ) | 5.341 |
| V (Ȧ^3^) | 135.717 | V (Ȧ^3^) | 136.19 | V (Ȧ^3^) | 136.35 | V (Ȧ^3^) | 152.358 |
|  |  |  |  |  |  |  |  |
| GDC |  |  |  | GDC phase |  | GDC phase |  |
| a=b=c (Ȧ) | 5.418 |  |  | a=b=c (Ȧ) | 5.392 | a=b=c (Ȧ) | 5.352 |
| V (Ȧ^3^) | 159.043 |  |  | V (Ȧ^3^) | 156.76 | V (Ȧ^3^) |  |
|  |  |  |  |  |  |  |  |
| LDC |  | LDC phase |  |  |  |  |  |
| a=b=c (Ȧ) | 5.515 | a=b=c (Ȧ) | 5.498 |  |  |  |  |
| V (Ȧ^3^) | 167.739 | V (Ȧ^3^) | 166.193 |  |  |  |  |

**Table S2**. Summary of the electrochemical performance (OCV, MDP) for the cells containing GDC and LDC buffer layer fabricated at 1250 ^o^C, respectively.

|  | GDC-1250 | | LDC-1250 | |
| --- | --- | --- | --- | --- |
| Tem  (^o^C) | O.C.V  (V) | M.P.D (W cm^-2^) | O.C.V  (V) | M.P.D (W cm^-2^) |
| 800 | 1.115 | 0.507 | 1.106 | 0.51 |
| 750 | 1.127 | 0.28 | 1.117 | 0.31 |
| 700 | 1.137 | 0.14 | 1.126 | 0.18 |


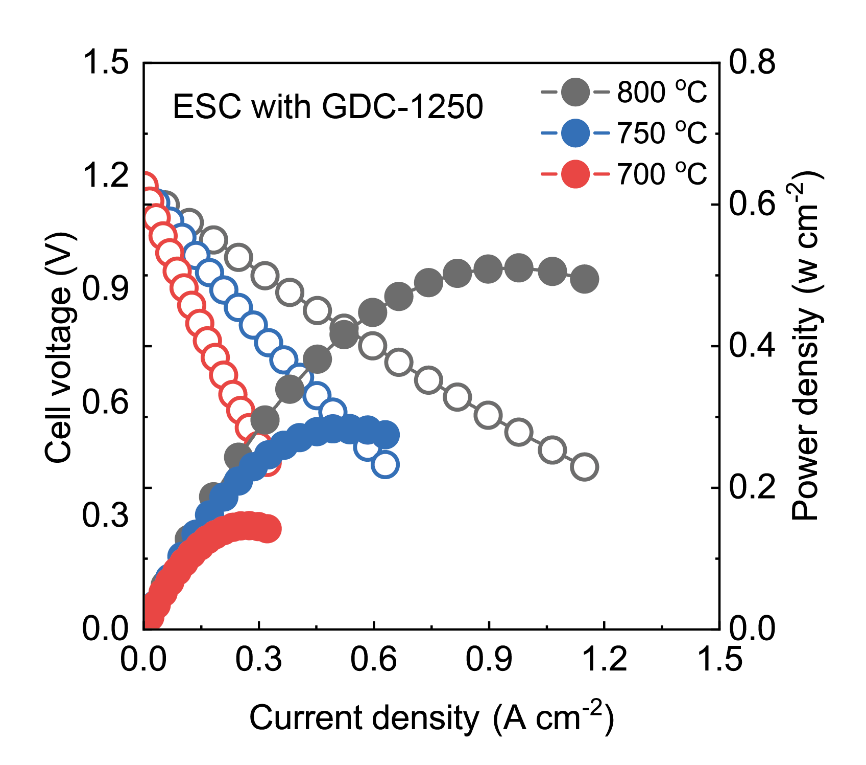


**Figure S3.** I-V curves of the electrolyte-supported cell (ESC) using GDC-1250 layer at 800, 750, and 700 ^o^C.


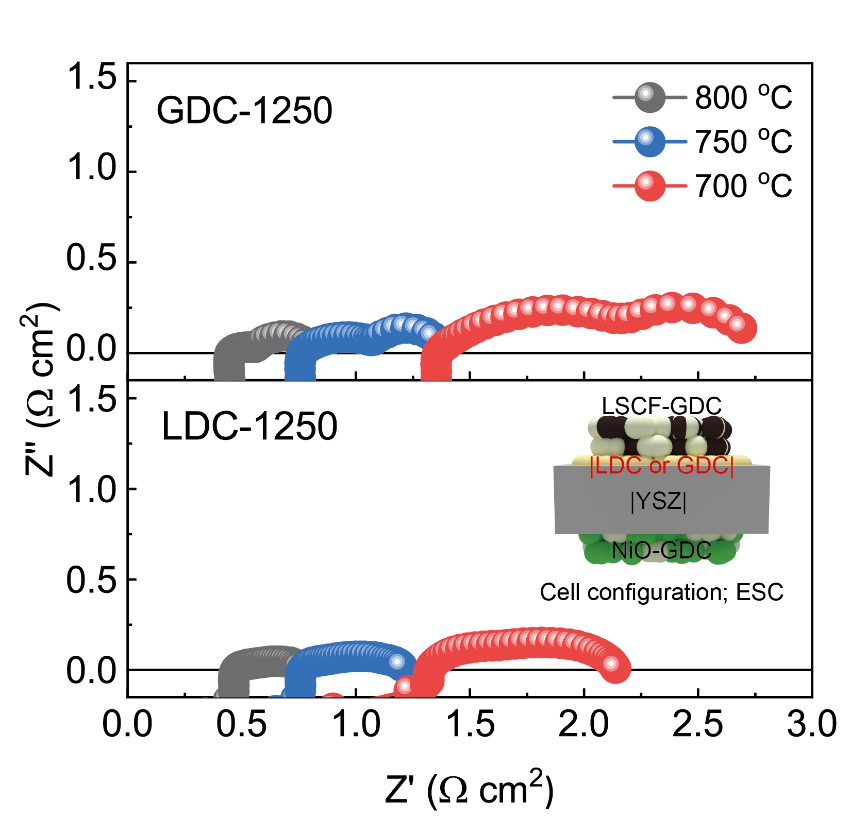


**Figure S4.** Impedance spectra of the ESC with GDC-1250 and LDC-1250 layer


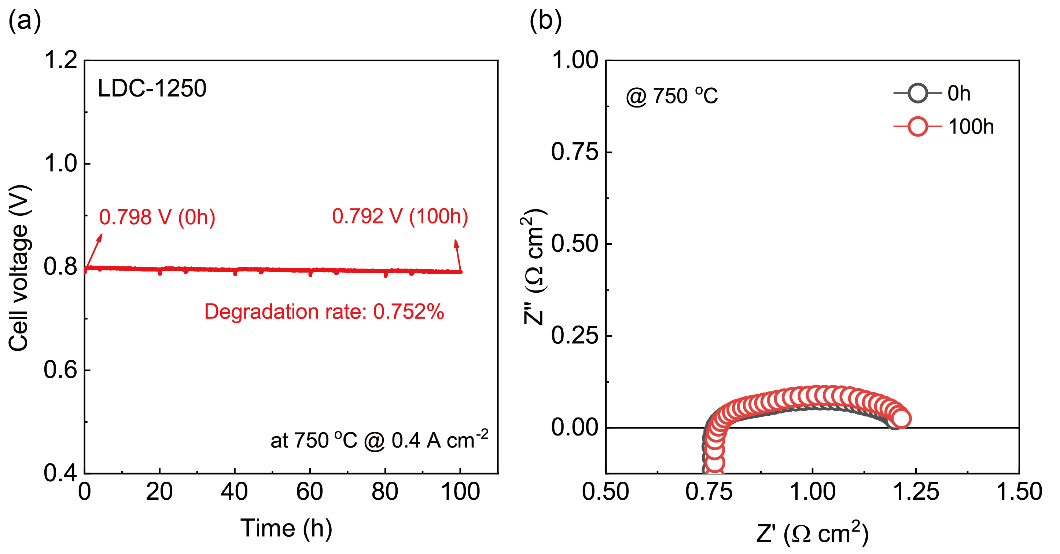


**Figure S5.** (a) The mid-term stability of the ESC with LDC 1250 layer. (b) Impedance results of the ESC with LDC 1250 layer before and after mid-term stability test.


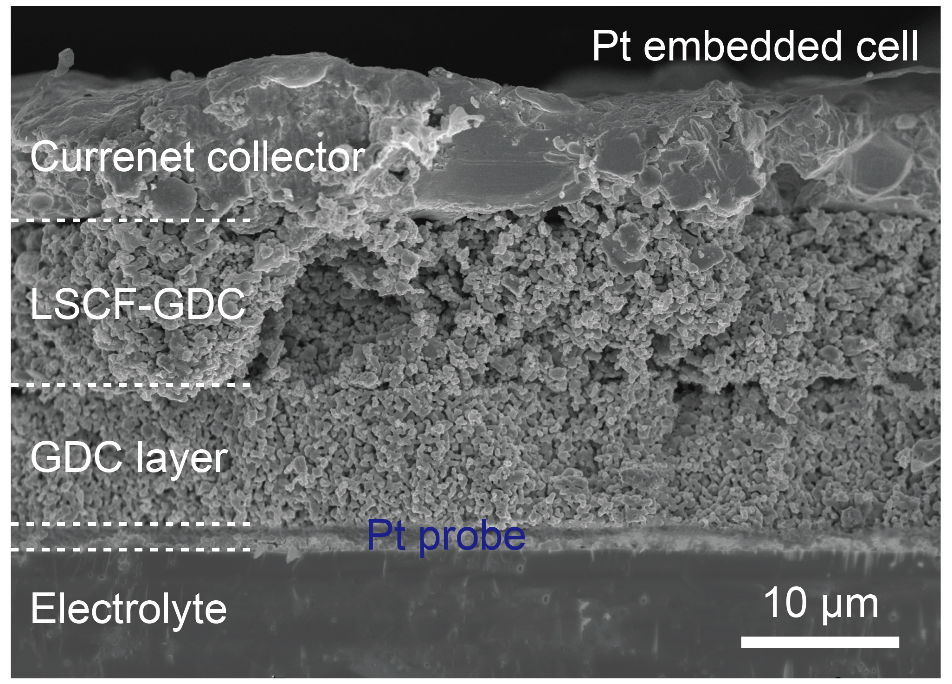


**Figure S6.** The cross-sectional SEM image of the Pt embedded cell with GDC layer


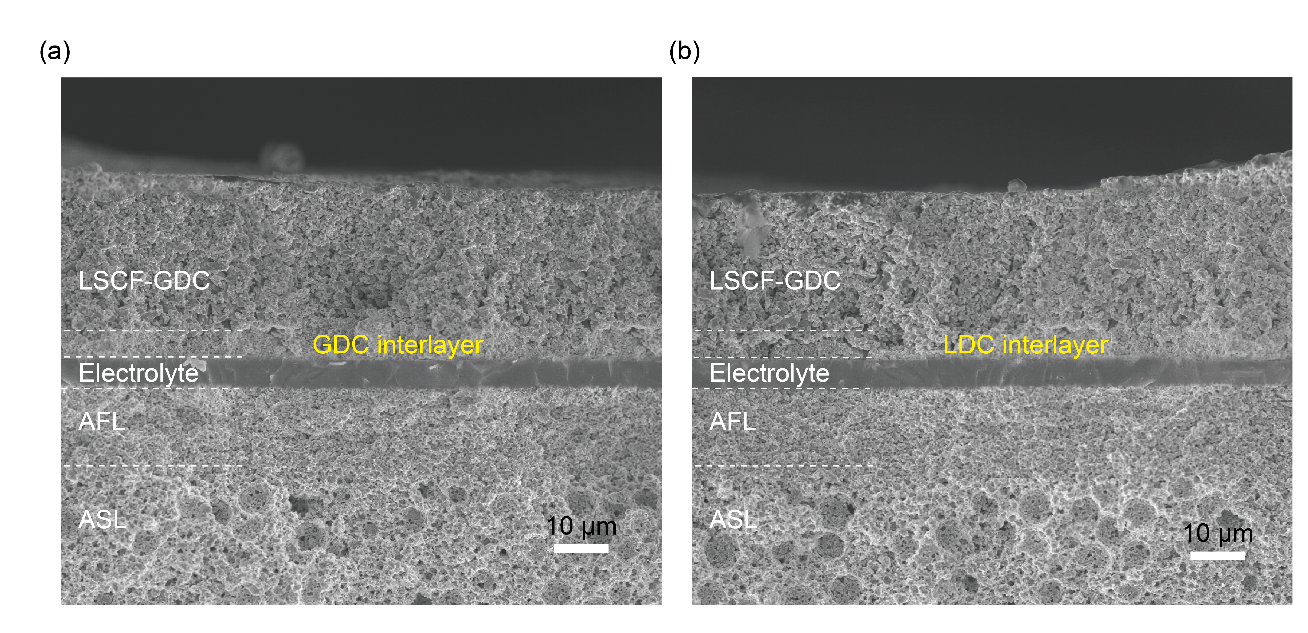


**Figure S7.** Cross-section SEM images of the ASC with GDC-1250 and LDC-1250 layer


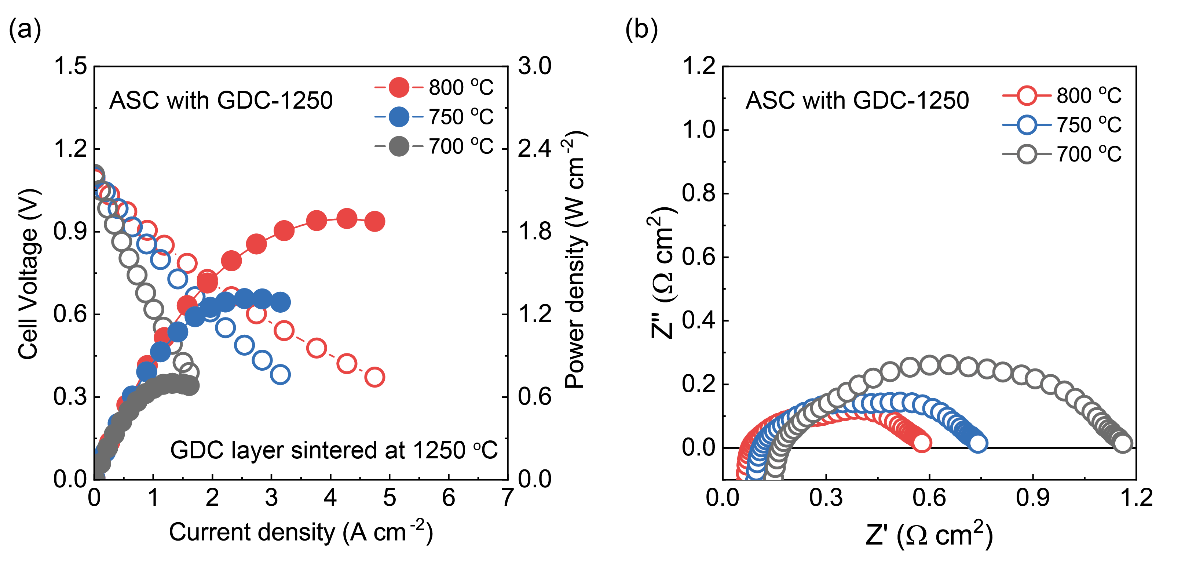


**Figure S8.** (a) I-V curves and (b) impedance spectra for the anode-supported cell (ASC) using GDC layer sintered at 1250 ^o^C.

**Table S3.** Summary of the R_s_ and R_p_ of the ASC with LDC and GDC layer applied bias; 0 A cm^-2^, 1 A cm^-2^, and 2 A cm^-2^.

|  | LDC | | | GDC | | |
| --- | --- | --- | --- | --- | --- | --- |
|  | 0 A cm^-2^ | 1 A cm^-2^ | 2 A cm^-2^ | 0 A cm^-2^ | 1 A cm^-2^ | 2 A cm^-2^ |
| R_s_ (Ω cm^2^) | 0.089 | 0.057 | 0.045 | 0.105 | 0.078 | 0.65 |
| R_p_ (Ω cm^2^) | 0.494 | 0.153 | 0.126 | 0.633 | 0.252 | 0.207 |


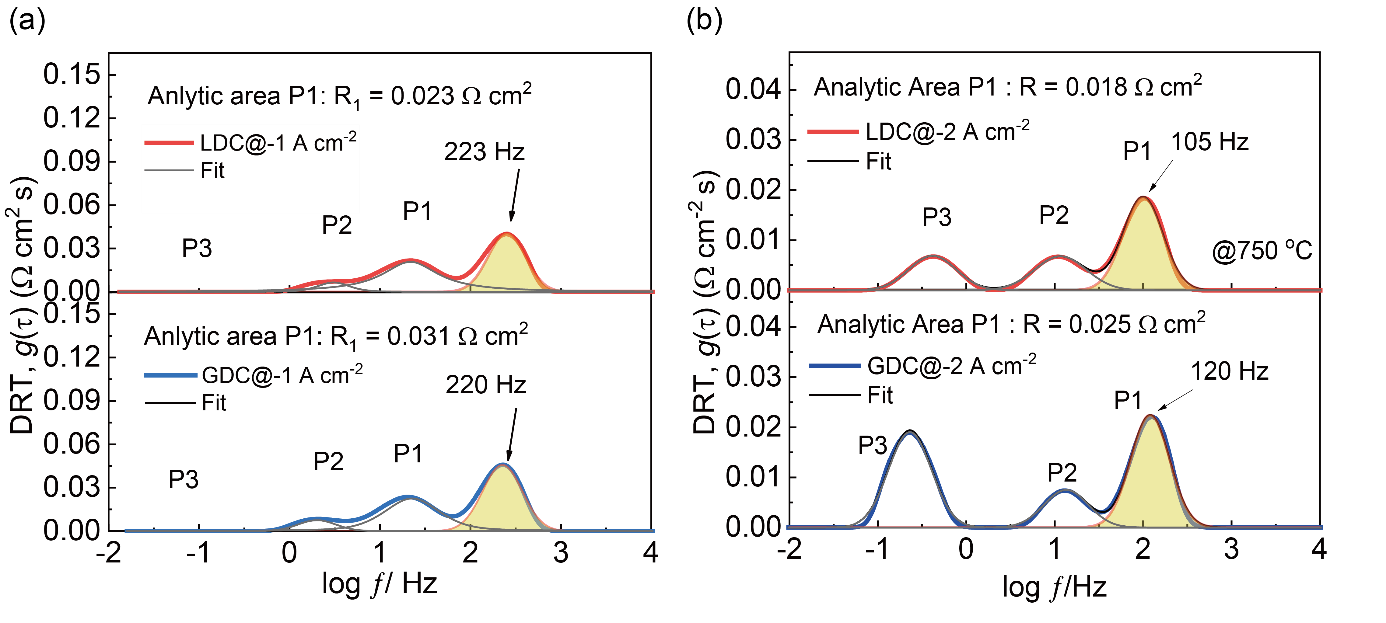


**Figure S9.** Distribution of relaxation time (DRT) plots of the ASC with LDC and GDC layer applied bias; (a) 1 A cm^-2^ and (b) 2 A cm^-2^.
